# Study of Effective Corridor Design to Improve Wayfinding in Underground Malls

**DOI:** 10.3389/fpsyg.2021.631531

**Published:** 2021-08-02

**Authors:** Shaoqing Zhang, Soobeen Park

**Affiliations:** Pusan National University, Busan, South Korea

**Keywords:** wayfinding, turn taking, built environment, virtual reality, virtual environments

## Abstract

In retail space, wayfinding difficulties can cause problems, such as loss of time, stress, or discomfort, negatively affecting the shopping experience of consumers and even their patronage intentions. Although studies have reported that space configuration may facilitate navigation, there has been a lack of detailed discussion, particularly in underground malls, where people often encounter wayfinding issues. In this study, a series of exit-finding tasks in virtual malls were simulated to determine if it was practical to encourage turn taking by changing the corridor width, length, height, or angle. The results showed that people have a right-turn preference during exit finding. Moreover, exit-finders mostly prefer taking the upward pathway *via* stairs followed by corridors with broader widths or curved corners, exhibiting visible and similar navigation effects. Shorter corridors have a visible but relatively small affinity. This study provides some empirical evidence of how the corridor configuration influences the turn taking of people and provides a theoretical reference for adding a guiding function to the spatial arrangement in underground malls.

## Introduction

Traffic congestion and ground space limitations highlight the need for more urban underground spaces (Broere, 2016; Lee et al., 2017). In some densely populated cities, underground malls (also known as underground streets) have started hosting daily commuting and amusements because they provide pleasant thermal comfort, convenient traffic, and low-cost rent (Zhou and Zhao, 2016). On the other hand, in windowless spaces (including underground malls or buildings with closed doors and sealed windows), the absence of an external view may cause some occupants to experience claustrophobic symptoms, such as feeling trapped and uneasy, losing a sense of control over their environment, or cardinal directions (Ringstad, 1994; Lee et al., 2017), which may lead to wayfinding difficulties (Yokoi et al., 2015).

Retailers in malls, or otherwise trying to improve wayfinding, face an interesting dilemma. The retailers want customers to stay longer in the mall to encourage impulse purchases, but exit-finding difficulties negatively influence the shopping experience of the customers and even degrade the reputation of the store (Dogu and Erkip, 2000). Through signage or decoration methods, such as adding words, signs, and lighting to the environment (cf., Hidayetoglu et al., 2012; Vilar et al., 2014), wayfinding issues can be addressed effectively. Nevertheless, they cannot be eradicated because these methods are only considered supporting features. They cannot compensate for an adverse spatial design, such as limited space size, form, or layout (Raubal and Egenhofer, 1998; Buchner et al., 2009; Marquardt, 2011). Moreover, these methods barely address the core issues of wayfinding management in malls, such as providing practical information while not burdening the architectural space (Dogu and Erkip, 2000).

Environmental psychological studies have revealed the navigational function of spatial information (Arthur and Passini, 2002), as they suggest that well-designed spaces provide efficient cues inherent to the environment, which can guide people subconsciously while guaranteeing a smooth experience of wayfinding (cf., Raubal and Egenhofer, 1998; Apelt et al., 2007). These investigations have been followed by some studies conducted in terms of image choices, such as floor plans and virtual environment (VE) screenshots or photographs (Buchner et al., 2009; Frankenstein et al., 2010; Wiener et al., 2012), to explore the preference of the pathfinders of different spatial configuration attributes during an emergency (Veeraswamy et al., 2011; Vilar et al., 2013) or in a maze (Buchner et al., 2009; Frankenstein et al., 2010; Veeraswamy et al., 2011; Wiener et al., 2012; Hsieh et al., 2018; Süzer and Olguntürk, 2018). These studies are good reference points for improving indoor wayfinding through architectural methods. On the other hand, the results of wayfinding in an emergency or maze may not apply to everyday wayfinding issues in underground malls. This is because people may feel completely different in underground environments than in aboveground or outdoors (Ringstad, 1994; Lee et al., 2017). Moreover, human strategies in everyday wayfinding are believed to be different from emergency escape (Symonds et al., 2017). In particular, during emergencies, such as power outages or fires, pathfinders tend to rely more on human information (e.g., following crowds) and environmental (e.g., emergency signs, lights, and maps) than spatial information (including space size, form, and layout; Golledge, 1999; Arthur and Passini, 2002; Symonds et al., 2017). In leisure wayfinding, however, a spatial strategy is considered the preferred strategy rather than a signage strategy, social strategy, or others (Arthur and Passini, 2002; Symonds et al., 2017).

This study examined whether it was practical to encourage turn taking by changing the corridor width, length, height, or angle using virtual roaming technology to simulate specific turns when walking through the corridors. Four spatial configuration attributes (width, length, height, and angle) were extracted from a literature review and combined into pairs to obtain 11 different T-type intersections. Subsequently, the 3D Studio Max (3Dmax) and Unreal Engine 4 (UE4) were used to produce virtual malls that included all the intersections, followed by a simulated series of exit-finding tasks of 124 college students. Finally, the route choice of the participants and their time spent in the decision area were combined to measure the wayfinding performance during turn taking.

## Wayfinding Access-Related Studies

Unlike navigation, wayfinding is a daily life process that may be as simple as moving from room to room or as complicated as escaping from a building (Raubal and Egenhofer, 1998; Dogu and Erkip, 2000). Clear designs of wayfinding are intuitive and nonverbal and help users access various spaces within a building, thereby reducing stress and increasing efficiency (Apelt et al., 2007). This section presents a systematic review of indoor wayfinding design studies and divides wayfinding access into three dimensions: signage, decoration, and spatial configuration.

### Signage and Decoration Methods

The addition of signs, including the posted maps, is considered an effective method to resolve wayfinding problems in existing environments. Designers are keen to post arrows, swords, pictograms, and even maps on ceilings or walls at various decision points.

**FIGURE 1:**
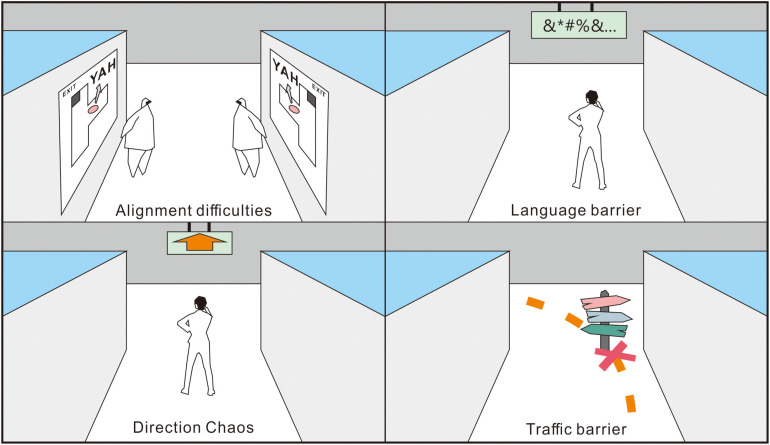
Potential issues with the use of signage strategies during wayfinding (cf., Levinew et al., 1984).

Balancing signage simplicity and regularity is considered problematic (cf., Levinew et al., 1984; Calori and Vanden-Eynden, 2015; Marquez et al., 2017). Calori and Vanden-Eynden (2015) suggested that effective signage must be legible from a distance, clear, and straightforward in design, have sufficient but not too much information, and be placed precisely where the travelers need information (e.g., at decision points). An obvious sign may also be confusing when placed in an arena of other signs competing for visual attention. Moreover, the challenge of designing comprehensible and iconic symbols for signs is particularly significant (Figure 1). For example, does an arrow pointing straight up mean “go forward” or “go up one floor?” Most nonprofessionals find it difficult to relate a 2D map (e.g., the “you are here” map) to directions in real 3D space (Levinew et al., 1984).

Focusing on these limitations in signage strategy, designers have attempted other ways to assist in wayfinding. Arthur and Passini (2002) reported that wayfinding does not need to be sign-upon-sign-upon-sign; they suggested that designers “use light and color before words, and then use words.” The differences between spaces are enhanced by adjusting the color or lighting (cf., Süzer and Olguntürk, 2018) or adding greenery (Tifferet and Vilnai-Yavetz, 2017), which in turn improves the understanding and memory of the people of their position. In other words, they pay more attention to the weighting of different nonspatial cues designed to provide wayfinding information.

For example, researchers conducting a study on hotel corridor design found that most participants selected directions with better lighting when lost because of their need for security (Vilar et al., 2013). Tifferet and Vilnai-Yavetz (2017) reported that the presence of greenery enhances the approach behavior of people. Lee et al. (2017) stated that it appears natural for people to logically link greenery to outdoor spaces, followed by some designers who used artificial plants as a landmark to guide people to the exits (Supplementary Figure 1). Although signage and decoration methods have alleviated wayfinding problems in underground environments, even the best-designed or best-placed signs and decorations cannot entirely compensate for the poor characteristics of architectural space. This is because the spatial configuration attributes (i.e., corridor width, length, height, and angle) have a relatively permanent nature–once space is built, they would hardly change in the short term (Buchner et al., 2009; Marquardt, 2011).

### Spatial Configuration and Wayfinding

Environmental psychology-related studies have suggested that that thoughts and feelings of people are shaped by the spaces they inhabit (Hall, 1966; Gehl, 2011; An et al., 2019). In an indoor environment (Supplementary Table 1), a corridor space comprises a ceiling, a floor, and two sidewalls that contribute the following four spatial attributes, namely: width, length, height, and angle. Among them, the adjustment of the corner curvature of the wall and the addition of steps to the floor are the most economical and effective means of changing the corner curvature and height, respectively. The four spatial configuration attributes of a corridor concern the human need for safety, privacy, public order, curiosity, and even authority, facilitating behavioral adjustments (Golledge, 1999; Barker, 2019) and have potential navigational functions.

#### Corridor Width

**FIGURE 2:**
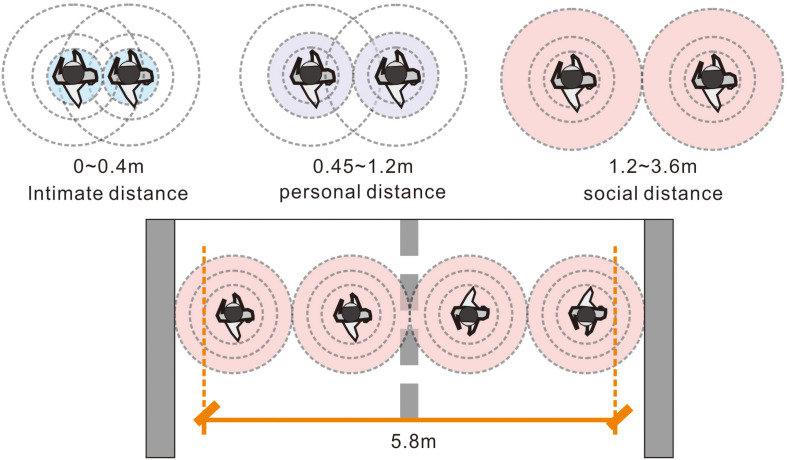
Relatively comfortable width of a two-way path (cf., Hall, 1966).

**FIGURE 3:**
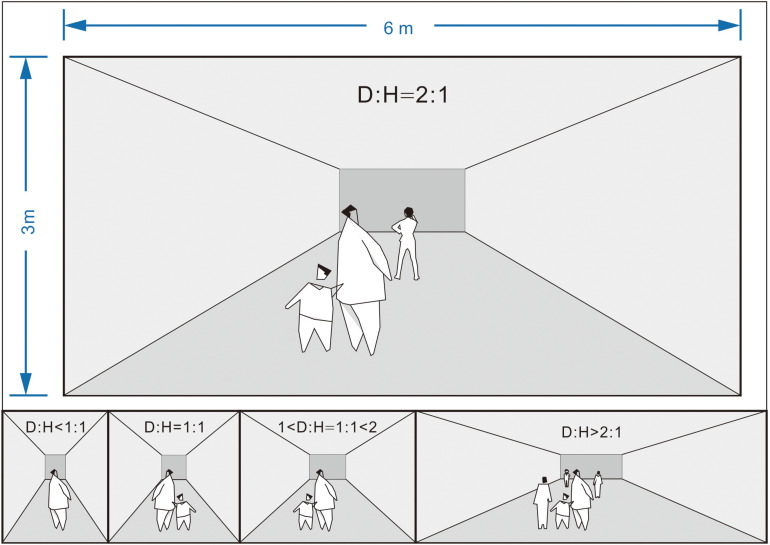
Relatively comfortable proportions of the corridor width and height (cf., Ashihara, 1985; Park and Zhang, 2019).

The corridor width is associated with privacy because it restricts the social distance among occupants. Hall (1966) found that the shortest distance a person can tolerate between their acquaintances is 4 ft (∼1.2 m). Once a stranger enters the area, an occupant may feel nervous and stressed and may even want to escape (Figures 2, 3). In an emergency escape (Sun and de Vries, 2013; Vilar et al., 2013), the width of the corridor influences the perceptions of safety, and broader corridors are considered safer. Vilar et al. (2013) examined the route choices of 30 students using five photographs of a computer-generated hotel (each photo simultaneously displayed two corridors with different widths). Before the experiment, the researcher stated, “The hotel is on fire now. You need to leave here as soon as possible.” The participants were then asked to choose one of the static images by pressing a left or right button. Approximately 70% of the participants chose the picture with the most expansive pathway.

#### Corridor Length

The corridor length influences the curiosity and desire of the pathfinders to explore, with longer corridors promising more information gain than alternative ones (Buchner et al., 2009; Frankenstein et al., 2010; Wiener et al., 2012). In a treasure-hunting simulation using a maze (Wiener et al., 2012), 20 adults searched for a gold bar among 30 screenshots that displayed a virtual space with different depths of view. As the participants performed the task, eye-tracking equipment recorded their sight focus. The results suggested that viewers stared more frequently at relatively longer paths. In another simulation to find the center and exit of a maze (Frankenstein et al., 2010), 21 adults engaged in a forced-choice task between two snapshots of a virtual maze (with each picture showing corridors with different lengths). Most participants preferred the longer path when the difference in length between two paths exceeded 10 m.

#### Corridor Height

A higher spatial position usually indicates safer conditions or higher social status, the same way that a podium functions in a classroom or a lecture hall (Gehl, 2011). In an outdoor environment, pedestrians and vehicles can be divided effectively by sunken squares or roadside platforms as pedestrians will consciously walk in higher places (Shaofei and Zhongwei, 2016). Niu (2009) reported that stair settings influence the route choice of an individual during vertical wayfinding. In that study, 46 adults observed the floor plan of a mall and chose a path between shop A (on the first floor) and shop B (on the second floor) with three routes of similar length (∼70 m) but different stair positions (near the start of the path in route 1, near the middle of the path in route 2, and near the end of the path in route 3). Approximately 76% of the participants chose route 1 to go upstairs. Li et al. (2019) also found the potential navigation function of stair settings. Specifically, the research was conducted in a two-story shopping mall, and the participants were asked to start from the second floor and arrive at the designated location on the first floor of the mall. As a result, most pathfinders tend to move vertically (via stairs) first rather than horizontally during wayfinding.

#### Corner Curvature

Smooth curves at intersection corners usually facilitate safe and quick turns. In an outdoor environment, building intersections usually have cut or rounded corners because pedestrians or drivers need sufficient sight distance to decide whether it is safe to turn. The corner curvature at junctions was suggested to be calculated according to the sight triangle principle, which depends on the reflection time and moving speed of the people (Supplementary Figure 2; Harwood et al., 1996; Easa, 2000). In an online survey involving floor plan images (Veeraswamy et al., 2011), 1,166 participants were instructed to imagine themselves escaping from a maze and needing to choose between two corridors with the same length but different corner types (curved versus orthogonal). Approximately 60% of the participants chose the path with a curved corner rather than an orthogonal corner, despite knowing that both routes have the same length.

As suggested above, several studies have used the route choice frequency to evaluate the wayfinding performance (an index that reflects the effectiveness of the wayfinding design; Supplementary Table 1). These studies predicted that the wayfinding performance improved with higher route selection proportions (Wiener et al., 2012; Vilar et al., 2013). On the other hand, a higher percentage of directional choices may not necessarily correspond to a better wayfinding performance. As Symonds et al. (2017) suggested, “although both walking and driving can reach the destination, the experience is entirely different.” Even if pathfinders choose the same route, they may differ in terms of their wayfinding performance because some choices may be firm, but others may be indecisive. Wayfinding is considered a complex cognitive process where the time spent is also a crucial issue. The process includes information processing, decision-making, and decision execution (Ruddle and Lessels, 2006; Süzer and Olguntürk, 2018). Accordingly, studies have suggested that two indicators determine the wayfinding performance during turn-taking: the route choice results that reflect the preferred route of an individual (Vilar et al., 2013) and the time costs (during decision-making) that influence the degree of preference (Ruddle and Lessels, 2006; Süzer and Olguntürk, 2018).

## Materials and Methods

### Modeling

Recent years have seen the rise of virtual roaming as an essential virtual reality (VR) technology branch, through intelligent hardware and open-source game engine platforms, such as UE4 or Unity 3D (Liang, 2015). Unlike studies that used images, virtual roaming focuses on human–space interactions, i.e., participants can move, jump, or turn freely in a virtual scene as if they were playing a game. VR technology is more suitable for a wayfinding simulation because it provides an immersive real-world experience for the experimental participants (Liang, 2015; Süzer and Olguntürk, 2018).

Based on a literature review (Section “Spatial Configuration and Wayfinding”), the four corridor configurations, i.e., width, length, height, and angle, were combined to generate 11 different intersections, and 3Dmax and UE4 were used to produce virtual underground malls that included all the intersections. With reference to the International Building Code (International Code Council, 2000), which is an essential tool that addresses both health and safety concerns for buildings, the corridor height shall not be less than 2.4 m in underground malls (typically, around 3 m), and the length of the corridor should be no less than twice its width. Accordingly, the spatial differences were increased to ensure that they were noticeable while keeping the corridor spaces within a reasonable range (Supplementary Table 2) to avoid discomfort. In essence, the aisle height was set to 3 m (approximately 10 ft); the narrowest corridor was set to 6 m (approximately 20 ft); the widest corridor was set to 8 m (approximately 26 ft); the shortest corridor was set to 11 m (approximately 36 ft); the longest corridor was set to 22 m (approximately 72 ft). The radius of the corner fillet was set to 3 m (approximately 10 ft).

To simulate a realistic wayfinding environment as much as possible and improve the probability of wayfinding information being recognized in a complex social environment (Iftikhar et al., 2020), some everyday items, including artworks (e.g., ceramics and paintings), crafts, and sundries, were arranged neatly in the shops on both sides of the corridors with customers leisurely walking or chatting with salespeople (Supplementary Figure 3). In particular (Supplementary Table 2), the population flow was controlled to less than 10 pedestrians/m^2^/minute (service level-A) to avoid retail crowding based on the level of service standard developed by Fruin (1971). In addition, to avoid glare due to the purely white walls, some green decorative panels were set to cover the walls, making the entire corridor look less monotonous. Moreover, the background sounds of the virtual malls (cf., Stewart et al., 2016) and lighting conditions (brightness and color temperature) were considered carefully to produce a comfortable atmosphere (cf., Şener Yılmaz, 2018).

### Experimental Conditions

#### Control Condition

Although the nonspatial cues in virtual malls have been controlled to avoid any effective wayfinding information besides the four spatial configurations, individuals may have an innate directional bias when facing a two-directional path. This type of directional clue (left or right) was suggested as a variable that affects the route choices of the participants (Veeraswamy et al., 2011). Therefore, it should be controlled before the experimental tests.

**FIGURE 4:**
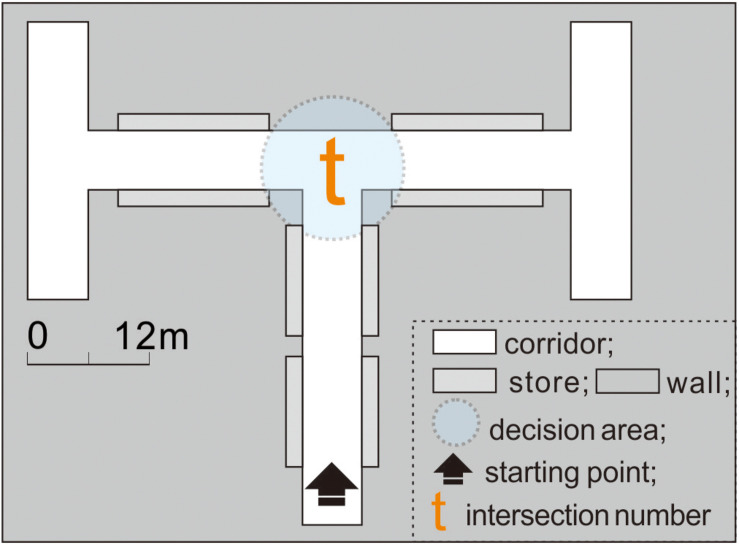
In experiment 1, intersection *t* showed no difference in the corridor configuration attributes between the left and right sides.

To examine if the participants in this study have a directional bias, experiment 1 was first conducted in the control condition environment (which showed the same corridor length, width, height, and angle between the left and right sides; Figure 4). All the turn-taking results of the participants and the time costs were recorded after each experiment.

#### Experimental Condition 1

**FIGURE 5:**
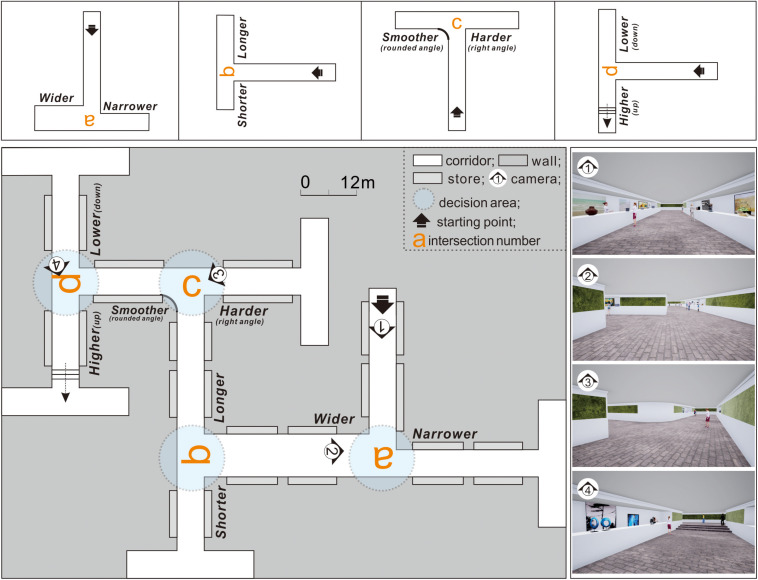
In experiment 2, intersections *a*–*d* showed differences in the corridor configuration attributes on one side of intersection *t*.

Experiment 2 was conducted under experimental condition 1 to examine if the turn-taking of exit-finders was influenced by changing the corridor width, length, height, or angle (Figure 5).

Taking intersection *t* as the standard, intersections *a*–*d* were created by separately adjusting the corridor width, length, height, and angle. For intersection *a*, the corridor width was increased on the right side (from 6 to 8 m). At intersection *b*, the length of the left corridor was reduced from 22 to 11 m. At intersection *c*, an arc with a 3 m radius was added to the corner of the left corridor. For intersection *d*, stairs were set on the left side corridor.

The side with different features remained the same during the experiment (e.g., the wider path was always on the right at the intersection during experiment 2). Otherwise, a comparison between the test results would be meaningless because the spatial and directional clues influence the results alternately.

#### Experimental Condition 2

**FIGURE 6:**
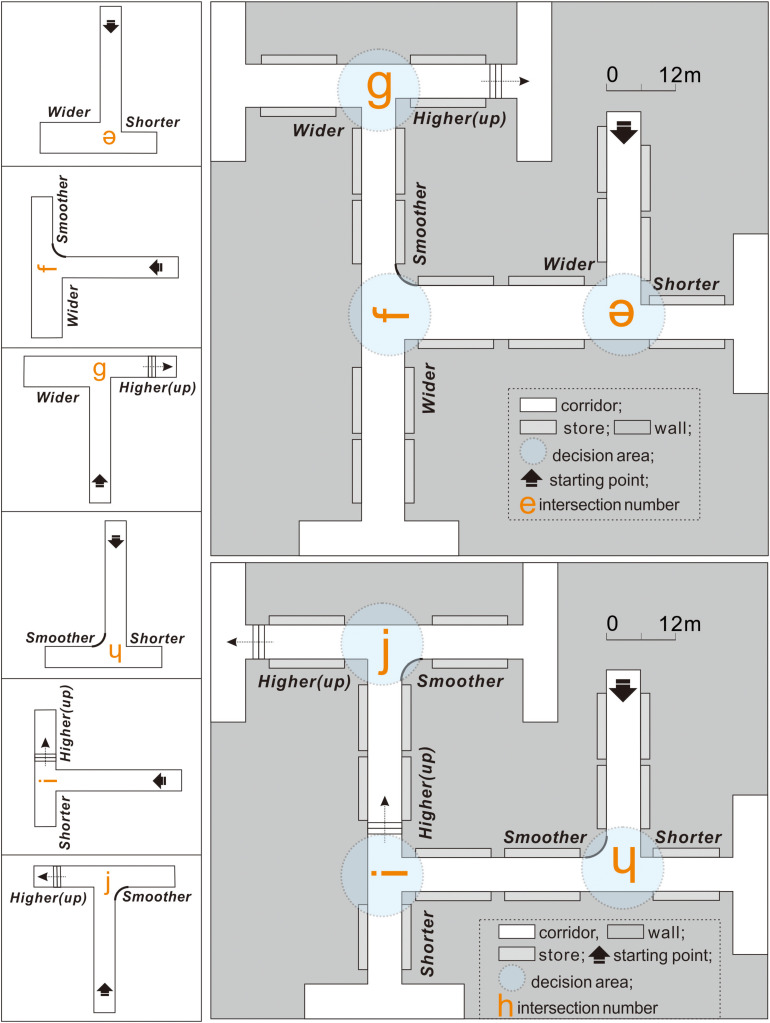
In experiment 3, intersections *e*–*j* have different corridor configuration attributes on both sides at intersection *t*.

Experiment 3 was conducted under experimental condition 2 (Figure 6), with various complex building environments to explore better corridor plans. Specifically, based on intersection *t*, the configurations on the left and right sides were changed simultaneously, and intersections *e*–*j* were obtained to compare each side with different features at intersections *a*–*d* in pairs, thereby obtaining spatial validity ranking information on turn-taking.

### Participants

G-Power 3.1.9.2.2 was used to obtain good statistical significance (G-Power is a tool to compute statistical power analyses for *t*-tests and *F*-tests), and a 0.5 effect size, a 0.05 α value, and a 95% confidence level were achieved. To properly implement the one-sample and paired *t*-tests, this study used a convenience sample comprising 124 college students, aged 18–32 years (half male and half female) from Pusan National University in Korea (*M* = 25.23, SD = 5.14). To improve the smoothness of the virtual experiments, the participants were required to have VR gaming experience when recruited. All the participants were recruited *via* campus e-mails and posts on the college bulletin board, and the wayfinding simulation experiments were conducted in the department laboratory. The study was approved and conducted in accordance with the standards of the Institutional Review Board of the university, and all participants completed an informed consent form before the study. Shopping vouchers were provided as an incentive for participation.

### Experimental Procedure

Owing to the potential influence of individual differences on the experimental results, the entire experiment followed a within-subject design to mitigate participant-to-participant variations (Gravetter and Forzano, 2018). This meant that each participant performed virtual wayfinding under both the control (Figure 4) and experimental conditions (Figures 5, 6). The experimental process was designed carefully to minimize the potential influences of the order effects (i.e., practice effects, fatigue effects, boredom effects, and carryover effects) as follows: (1) Familiarization with the process: Before conducting the experiments, the research purpose, time cost, and procedures were elucidated through a simple 3D game to help participants familiarize themselves with the VR equipment. (2) Simplifying the steps: To prevent the resistance of the participants due to cumbersome experimental processes, the 11 intersections were integrated into three virtual scenes (experiments 1–3; Figures 4–6, respectively). The experiments were conducted in order. (3) The use of counterbalancing: The turn-taking tests could start from any intersection in the same experiment. That is, the test sequence was not restricted by the intersection number. (4) Setting the interval: After each experiment, the participant was instructed to rest for at least 5 min to prevent the previous experiment from interfering with the next one.

As the experiments started, the scenario to each participant was described as follows: “You have finished shopping and need to leave the underground mall. Please find the exit according to your judgment of the spatial configurations of each intersection.”

Without external distractions (through blueprint coding, the game system will automatically prompt the participants when the test starts, how to proceed, and when it ends; Supplementary Figure 4), the participants used a joystick combined with VR head-mounted equipment to perform the wayfinding task in the following sequence: (1) The participants started walking along the corridor first. (2) As they entered a decision area with a 6 m radius, the background program began timing their decision-making. (3) The participants observed and compared the spatial information of the two paths and then decided on an action. (4) The timer ended when the participants left the decision area and recorded the time within an accuracy of 0.1 s. (5) The participants saw each intersection once only.

During the experiments, the time costs of the participants were recorded using the blueprint function of UE4 (written in C++) (cf., Zhang et al., 2018). Specifically, the decision area was a transparent “box trigger” with an added timer function, and two commands were given (cf., Supplementary Figure 5) as follows: (1) The timer started running when the pathfinder entered the decision area by touching the edge of the trigger for the first time. (2) When the pathfinder exited the area by touching the edge of the trigger again, the computer automatically recorded the route choice result and the time spent in the decision area.

### Analysis

When pathfinders faced a bidirectional path decision, their left- or right-turn preference should be complementary in theory (Raubal and Egenhofer, 1998). Simply put, the likelihood of turning right decreased with increasing preference to turn left. To facilitate understanding and statistical calculations, 100% was used as the maximum wayfinding performance score to indicate when pathfinders turned right or left with the least hesitation (time spent in the decision area was used to reflect the degree of hesitation, and a score of 100% corresponds to the participant who spent the least time from entry to leaving the area). A score of 50% indicated the minimum score of the left or right wayfinding performance, indicating that the participants were quite hesitant and experienced difficulties in choosing (cf., Supplementary Figure 6).

Considering the inverse relationship between the time spent and wayfinding performance (Ruddle and Lessels, 2006; Süzer and Olguntürk, 2018), if *t* is the time spent in the decision area, then the wayfinding performance *p* can be expressed as follows (1):

(1) p=-a*t+b

After all the 11 virtual tasks (one control condition and all the experimental conditions), 120 cases (1,320 total indicators) remained after removing the outliers and technical errors. The time that the participants spent in the decision-making area ranged from 3.5 to 14.6 s. Hence, the two extreme points “i” (3.5 s, 100%) and “ii” (14.6 s, 50%) were substituted into formula (1), and *a* = -0.045 and *b* = 1.1577 were obtained (Supplementary Figure 7). The wayfinding performances of the participants in each intersection (regardless of the left or right turn) were then calculated. Finally, to facilitate statistical calculations, the “right-turn performance” was used for data analysis. For those who turned left, their right-turn performance was 100% minus the version of a left turn.

### Hypotheses

Based on the literature in Section “Spatial Configuration and Wayfinding”, five hypotheses were formulated to explore the relationship between the corridor configuration attributes (width, length, height, and angle) and turn taking of the exit-finders:

H0: At intersection *t*, the exit-finders prefer to turn right.
H1: At intersection *a*, the exit-finders prefer to turn right (wider path).
H2: At intersection *b*, the exit-finders prefer to turn right (longer path).
H3: At intersection *c*, the exit-finders prefer to turn left (path with a curved corner).
H4: At intersection *d*, the exit-finders prefer to turn left (path with steps on the floor).

A one-sample *t*-test was conducted to verify the tendency of the participants to turn right after the control condition experiment (experiment 1). A paired sample *t*-test was performed after experiment 2 to determine if the configuration of the intersection, rather than the innate left- or right-turn preference of the participants, affected their turn taking. A paired sample *t*-test was conducted after experiment 3 to compare each side with different features at intersections *a*–*d* in pairs, thereby obtaining spatial validity ranking information on turn taking.

## Results

### Right-Turn Preference of Participants During Exit-Finding

**FIGURE 7:**
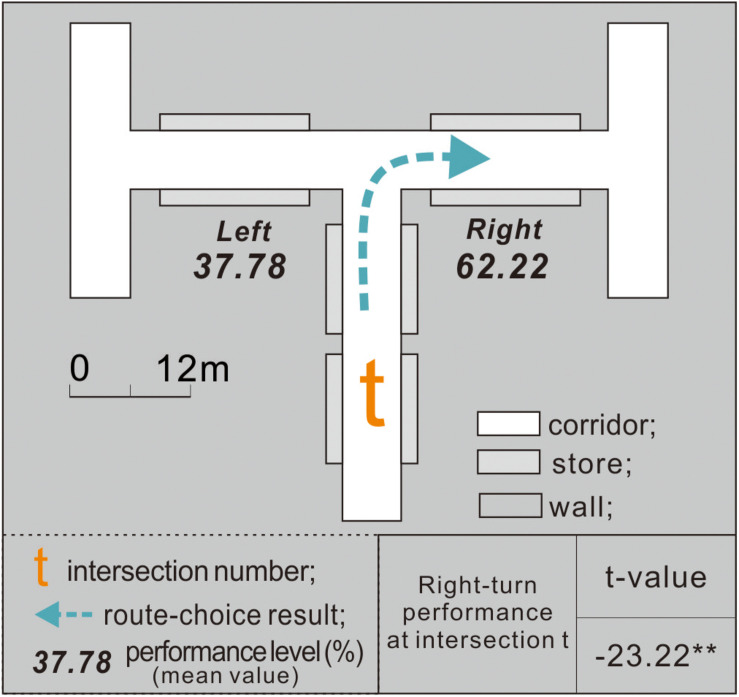
Exit-finders had an apparent right-turn preference at intersection *t*.

When the participants finished the wayfinding task under the control condition (experiment 1), the right-turn performance of all 120 participants at intersection *t* was calculated using formula (1). The results in Figure 7 indicate that at intersection *t*, with the same corridor configuration attributes, the exit-finders exhibited a mean right-turn performance of 62.22% (>50%). This suggests that the participants may have preferred to turn right while attempting to find an exit; those who turned right spent less time in the decision area than those who turned left.

The one-sample *t*-test confirmed that at intersection *t*, the participants exhibited an apparent right-turn tendency while looking for an exit (*p* = 0.05). Therefore, H0 was supported.

### Participants Preferred a Taller, Wider, Smoother, and Shorter Path

**FIGURE 8:**
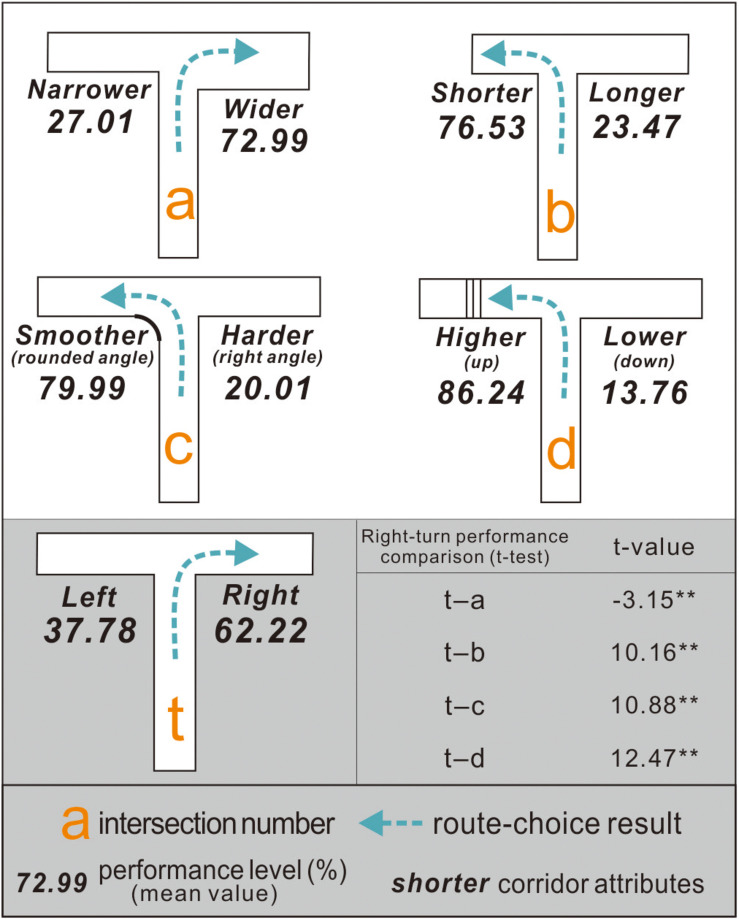
Right-turn performance of exit-finders at intersections *a*–*d* indicated different preferences from intersection *t*.

After experiment 2, the right-turn performance of the 120 participants at intersections *a*–*d* was computed. To illustrate (Figure 8), for intersection *a*, the corridor width was adjusted on the right side (from 6 to 8 m), which increased the right-turn performance of the participants (mean value) from 62.22% (at control condition intersection *t*) to 72.99%. Such an increase in the right-turn performance indicated that broader corridors increased the original preference of exit-finders for right turns. At least three factors may have contributed to an increase in right turn performance: (1) the number of participants who turned right increased; (2) the number of participants who turned right did not change, but the participants who turned right spent less time at intersection a than at intersection *t*; (3) both the number of participants who turned right and their time spent did not change, but the participants who turned left were more hesitant and took longer to make a decision.

At intersection *b*, when the length of the left corridor was reduced from 22 to 11 m, the right-turn performance of the exit-finders decreased from 62.22 to 23.47% (<50%). This means that a shorter corridor disrupted their original preference for turning right and increased their likelihood of turning left. While some maze design studies have found that people prefer longer or deeper corridors when finding exits (Buchner et al., 2009; Frankenstein et al., 2010; Wiener et al., 2012), these results indicated that this theory is unsuitable for situations similar to the virtual underground malls here.

At intersection *c*, an arc with a 3 m radius was added to the corner of the left corridor, which decreased the right-turn performance of the participants from 62.22 to 20.01%. Unlike the case in intersection *t* (control condition), the stairs on the left side of intersection *d* were set. As a result, the average right-turn performance of the exit-finders was reduced further to 13.76%, as they finally chose to turn left despite their original right-turn preference.

The *t*-test results of all the experimental conditions (*a*–*d*) and control conditions exhibited significant differences (*p* = 0.05). This finding supported H1, H3, and H4 but not H2.

### Participants Preferred the Upward Pathway via Stairs the Most

**FIGURE 9:**
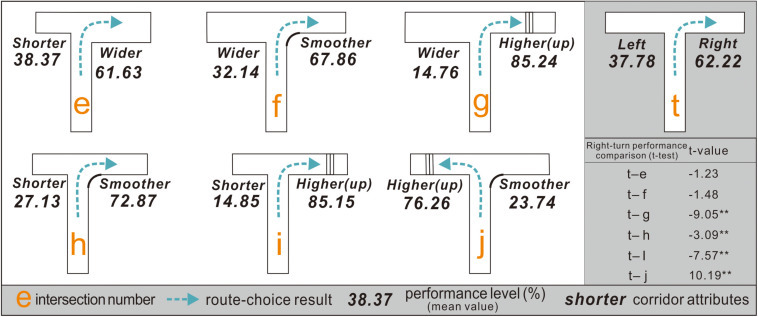
Right-turn performance of exit-finders at intersections *g*–*j* demonstrates differences from that at intersection *t* but not for intersections *e* and *f*.

After experiment 3, the right-turn performance of each participant was calculated separately at intersections *e*–*j* (Figure 9): (1) At intersection *e*, the participants had a right-turn preference with a mean performance value of 61.63%. (2) At intersection *f*, their right-turn performance was 67.86%. (3) At intersection *g*, the participants preferred to turn right, with a mean performance of 85.24%, which indicated that people might prefer a taller corridor to a wider configuration. (4) At intersection *h*, the participants chose to turn right with a mean performance of 72.87%. This indicated that exit-finders were more likely to choose a smoother corridor to a shorter configuration. (5) At intersection *i*, the participants preferred to turn right, with a mean performance of 85.15%, suggesting that people were more inclined toward a taller corridor over a shorter configuration. (6) At intersection *j*, the participants chose to turn left with a mean performance of 76.26%.

The results of the paired sample *t*-test between the experimental condition intersections (*e*–*j*) and control condition intersection *t* indicated that the right-turn performance of the participants at intersections *e* and *f* was similar, suggesting that they had difficulty choosing between shorter and wider corridors, and between wider and smoother corridors. Nevertheless, further research is warranted because some participants stated that they hardly noticed the width discrepancy in the two routes despite their 2-m difference.

## Discussion and Conclusion

Overall, this study addressed two issues to improve wayfinding at T-type intersections in underground malls: whether the turn taking of exit-finders was influenced by adjustments in corridor configurations, i.e., width, length, height, or angle, and how much these spatial configuration attributes influenced the turn-taking decisions of the exit-finders and enhanced the practicality of the study.

This study showed that the exit-finders had an apparent right-turn preference when facing a T-type intersection. This kind of directional bias during exit finding appears to be a universal factor. Veeraswamy et al. (2011) once used online questionnaires combined with maze pictures to obtain feedback from 1,166 participants from 36 countries, where 67.1% believed that the exit might be on the right side of the T-type intersection. On the other hand, no further experimental research or discussion has been provided. Although the wayfinding simulation conducted using images lacked consistency, these results support the following findings. (1) Experiment 1 indicated that those who eventually chose to turn left at the T-type intersection were more hesitant, and they spent more time in the decision-making area than those who chose to turn right. In contrast, those who turned right were more confident about turn taking. The cause of this right-turn tendency could not be determined because the participants in this study were recruited *via* random sampling. On the other hand, according to Veeraswamy et al. (2011), there may be a cultural component (left- or right-hand traffic) to wayfinding, and according to Shaki et al. (2009), the reading direction, i.e., whether a culture reads from left to right or right to left, may influence the spatial representation of the individual. Hence, further research will be needed. (2) The route choices of the exit-finders can be modified by adjusting the corridor length or width, setting stairs, or adding an arc at the corner. In particular, exit-finders in malls tend to link the exit path logically with “taller,” “wider,” “smoother,” or “shorter” features. Experiment 2 indicated that exit-finders preferred the shorter path to the longer path in situations, such as intersection *b*, which is a departure from previous views. This is because Frankenstein et al. (2010) and Wiener et al. (2012) stated that when people were lost in a maze, they preferred a longer or deeper path to obtain more wayfinding information. These studies indicated that pathfinders had different preferences for spatial attributes in different locations, even when they had the same goal (e.g., finding an exit or an office room). (3) Exit-finders had a stable preference for upward pathway with visible stair settings (near the decision area that can be noticed), regardless of the wider, shorter, or smoother opposite path, followed by corridors with broader width or rounded angles, with shorter corridors exhibiting visible but relatively small affinity. A visible difference in height between corridor spaces appears to be important information (directional clue or otherwise) when pathfinders are looking for an exit. Accordingly, the challenge faced by designers or managers is the exploration of how to use the space design methods in addition to stairs to imply “you can go up from here.” Furthermore, the intersection layout in underground malls should avoid intersections *e* and *f*. Although exit-finders are more inclined to turn right than left at intersections *e* and *f* (Figure 6), the spatial information did not provide a practical navigational function.

Our findings have also some limitations, as they are based on a single experimental set in one specific country, conducted in a virtual reality simulation, in a specific behavioral setting (i.e., the mall), and with specific subjects (students). To enhance the external validity of our findings, future replications are needed using different participants (i.e., not only students but also adults or elderly people), different countries and cultures, different settings (such as, for example, residential buildings, offices, hospitals, metro stations, outdoor spaces, green areas, sport stadiums, and theaters, etc.).

## Implications

If the exit paths in underground shopping malls simultaneously fulfill the features of being “wider,” “shorter,” “taller,” and “smoother,” they will be considered “more attractive.” In other words, people will find these exits more natural and efficient. In practice, however, it is difficult for the corridor configurations of underground malls to fulfill all four requirements due to the constraints of the built environment or other factors. Therefore, mall managers need to make optimal choices according to their judgment.

Based on these results, this paper provides the following suggestions. First, creating visible spatial differences in a vertical direction, such as setting steps, slopes, or other exit path facilities, is important for guiding people toward exits. Steps or slopes need to be near decision points within the sight of people. The usability of stairs should also be considered, for example, whether they have gentle slopes and handrails to facilitate passage for the elderly or disabled individuals. In cases where the existing environment does not allow steps to be added, other architectural methods, such as the addition of vaulted ceilings or atrium spaces that introduce natural light, may be employed to remind people that they can go up from here. Second, visually shorter corridors also have an apparent guiding function associated with the time cost. Generally, setting exits relatively close to intersections helps improve wayfinding performance. If building structure restrictions require an exit to be located far from a decision area, an autowalk could be installed, or some horizontal or vertical structures could be added, such as beams or pillars, to divide the passage into segments, which can reduce the stress caused by long distances. Third, the width of an exit corridor should be increased to help pathfinders find exits more efficiently and prevent accidents, such as stampedes, during an emergency. If the width of a corridor is fixed and cannot be changed, the path space can be expanded by adding a curved corner at the intersection, and reflective metals or panels can be used for corridor decoration. Finally, setting a specific arc at the intersection corner also helps guide people toward an exit, as curved wall corners can help improve the sight of the pathfinders, thereby improving their perception of safety and confidence when turning.

Although these recommendations are not fully applicable to aboveground buildings, they may have specific reference value for improving the spatial layouts of hospitals, schools, and nursing homes. For example, at T-type intersections, if the exit follows a higher, shorter, wider, or smoother direction according to the spatial preferences of the exit-finders, the opposite direction will inevitably be “unpopular” or “unreachable” but will increase spatial privacy. A corridor in a direction opposite to the exit is appropriate when organizing spaces, such as lounges, reading rooms, and other areas requiring privacy and silence. In contrast, a corridor in the direction of an exit is more appropriate for planning relatively popular spaces, such as entertainment and conference rooms, thereby ensuring that crowds can evacuate in the event of a wayfinding problem.

## Data Availability Statement

The original contributions presented in the study are included in the article/Supplementary Material, further inquiries can be directed to the corresponding author.

## Ethics Statement

The studies involving human participants were reviewed and approved by Pusan National University Institutional Review Board, PNU IRB. The patients/participants provided their written informed consent to participate in this study.

## Author Contributions

SZ and SP have made substantial contributions to the conception or design of the work; or the acquisition, analysis, or interpretation of data for the work. SZ have drafted the work or revised it critically for important intellectual content. Both authors have approved the final version to be published, we agree to be accountable for all aspects of the work in ensuring that questions related to the accuracy or integrity of any part of the work are appropriately investigated and resolved.

## Conflict of Interest

The authors declare that the research was conducted in the absence of any commercial or financial relationships that could be construed as a potential conflict of interest.

## Publisher’s Note

All claims expressed in this article are solely those of the authors and do not necessarily represent those of their affiliated organizations, or those of the publisher, the editors and the reviewers. Any product that may be evaluated in this article, or claim that may be made by its manufacturer, is not guaranteed or endorsed by the publisher.

## Supplementary Material

Supplementary Figure 1 Green elements act as landmarks to guide exit-finders in interior space (cf., Lee et al., 2017).

Supplementary Figure 2 Curved corners improve driver confidence when cornering or going straight (cf., Easa, 2000).

Supplementary Figure 3 Details of the operational procedures.

Supplementary Figure 4 Blueprint code used to guide the wayfinding simulation.

Supplementary Figure 5 Blueprint timing function code of UE4.

Supplementary Figure 6 50% is a theoretical threshold between left- and right-turn performance (Raubal and Egenhofer, 1998).

Supplementary Figure 7 Time cost versus right-turn performance.

Supplementary Table 1 Wayfinding empirical studies involving corridor configurations.

Supplementary Table 2 Specific parameters of the virtual scene.
