# Supplementary material for: Study of Effective Corridor Design to Improve Wayfinding in Underground Malls
Source: Front Psychol. 2021 Aug 2;12:631531. doi: 10.3389/fpsyg.2021.631531 (PMC8364955; doi:10.3389/fpsyg.2021.631531)
Supplement: Supplementary Figure 1 — Green elements act as landmarks to guide exit-finders in interior space (cf., Lee et al., 2017). [file Table_1.DOC]

Table S1. Wayfinding empirical studies involving corridor configurations

| Authors | Virtual environment | Settings | Samples | Tools | Analysis methods | Main findings |
| --- | --- | --- | --- | --- | --- | --- |
| Buchner et al. (2009) | 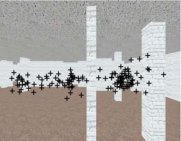 | Virtual maze. Two corridors with the same depths of view. | 20 adults | 30 screenshots | Compared subjects’ sight focus, which was recorded using eye-tracking equipment. | Pathfinders were more inclined to choose the direction with a relatively longer view. |
| Niu (2009) | 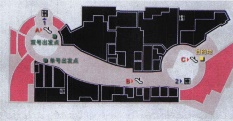 | Virtual mall. Three routes with the same length but different stair-setting positions. | 46 adults | Three floor plan images | Manually recorded and compared subjects’ choice frequency for the three routes. | More than 70% of the subjects chose the path with the closest step setting. |
| Frankenstein et al. (2010) | 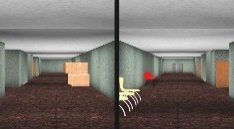 | Virtual maze. Two corridors with different lengths. | 21 adults | Five screenshots made using Google SketchUp | Manually recorded and compared subjects’ choice frequency for the routes. | When the length difference between two corridors exceeded 10 m, the subjects preferred the longer one. |
| Veeraswamy et al. (2011) | 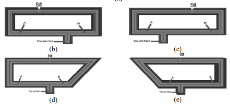 | Virtual maze. Two corridors with the same length but different corner types (curved versus orthogonal). | 1,166 participants from 36 countries | Online survey combined with floor plan images | Compared subjects’ choice frequency for the two paths. | Around 60% of the subjects would choose the path with curved corners during emergencies. |
| Wiener, et al. (2012) | 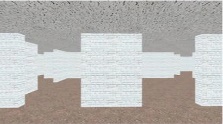 | Virtual maze. Two corridors with different depths of view. | 20 adults | 30 screenshots | Compared subjects’ sight focus, which was recorded using eye-tracking equipment. | Pathfinders were more inclined to choose the direction with a relatively longer view. |
| Vilar et al. (2013) | 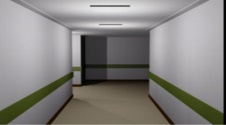 | Virtual hotel. Five corridors with widths ranging from 2–4 m. | 30 college students | VR photos made using [3Dmax](https://baike.baidu.com/item/3D Studio Max) | Manually recorded and compared subjects’ choice frequency for the five routes. | About 70% of the subjects would choose the widest path in an emergency. |
| Sun and de Vries, (2013) | 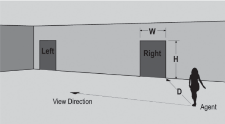 | Forty-nine virtual scenes, each has two exit doors with different withs | 187 college students | Automatic virtual environment | Compared subjects’ choice frequency for the doors. | The attribute width is an indispensable factor in the selection. |
| Süzer and Olgunturk (2018) | 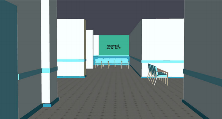 | Three virtual mazes with different colors (cool, warm and neutral colors). | 20 people ages from 55-73 | Sketch Up | Compare the time required for the participants to find the exit. | Older people perform better in cool-toned wayfinding. |
| Hsieh et al. (2018) | 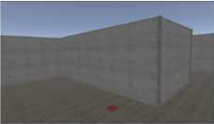 | A virtual maze | 70 college students | Virtual roaming based on Unity 3D | Compare the experimental efficiency of two virtual roaming tools (VR glasses vs. mouse & keyboard). | Keyboard & mouse performed good at wayfinding efficiency. VR has better sense of spatial orientation. |

Table S2. Specific parameters of the virtual scene

| Category | Item | Parameter | Comments | Reference |
| --- | --- | --- | --- | --- |
| Parameters of the virtual malls | Corridor width | Narrowest 6 m (20 ft), widest 8 m (26 ft) | Six m is a relatively comfortable width of a two-way path. | International Code Council. (2000); Hall, (1996); Ashihara, (1985) |
| Corridor length | Shortest 11 m (36 ft), longest 22 m (72 ft) | A length difference that is easily perceptible visually. Generally, the corridor’s length is not less than twice its width. | Frankenstein et al. (2010), International Code Council. (2000) |
| Ceiling height | 3 m (9.8 ft) | The height shall not be less than 2.4 m in underground malls; 3 m is a common height. | International Code Council. (2000) |
| Floor height | Four steps, a total of 0.5 m (1.64 ft) high | Set within a clearly visible range. | Niu (2009) |
| Corridor angle | The radius of the corner fillet is 3 m (9.8 ft) | A safe corner area according to walking speed and field of view. | Easa (2000), Harwood et al. (1996) |
| Ambient sound | 60 dB | Ambient sounds from a shopping mall were played. | Stewart et al. (2016) |
| Illumination | 700 lux | According to lighting standards, this is a relatively bright lighting condition that does not easily cause depression. | Boyce (2014), Şener (2018) |
| Lighting temperature | 5,000 K |
| Population flow | 10 avatars/m^2^/minute | The level-A service standard (less than 23 pedestrians/m^2^/minute) | Hall (1966); [Fruin (1971)](http://www.sciencedirect.com.ssl.eproxy.pusan.ac.kr/science/article/pii/S027249441930043X?via%3Dihub#bib12); Park and Zhang (2019) |
| Virtual perspective | Moving speed | 1.2 m per second | The average walking speed in real time. | – |
| View height | 1.6 m (5.2 ft) | The average visual height of subjects. |


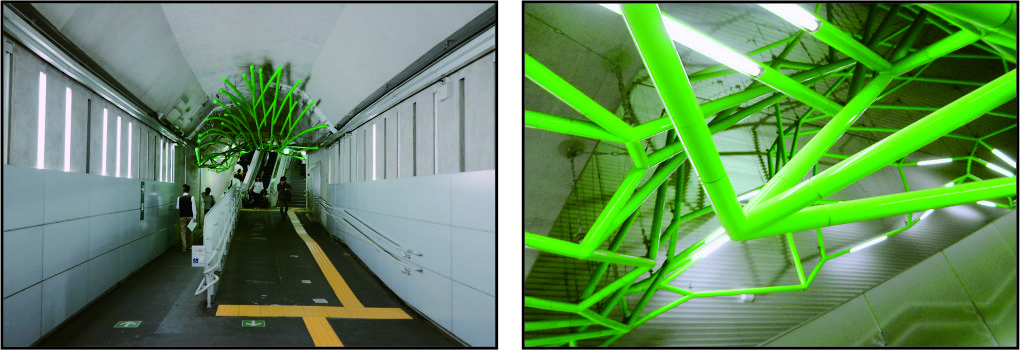


Figure S1. Green elements act as landmarks to guide exit-finders in interior space (cf., Lee et al., 2017).


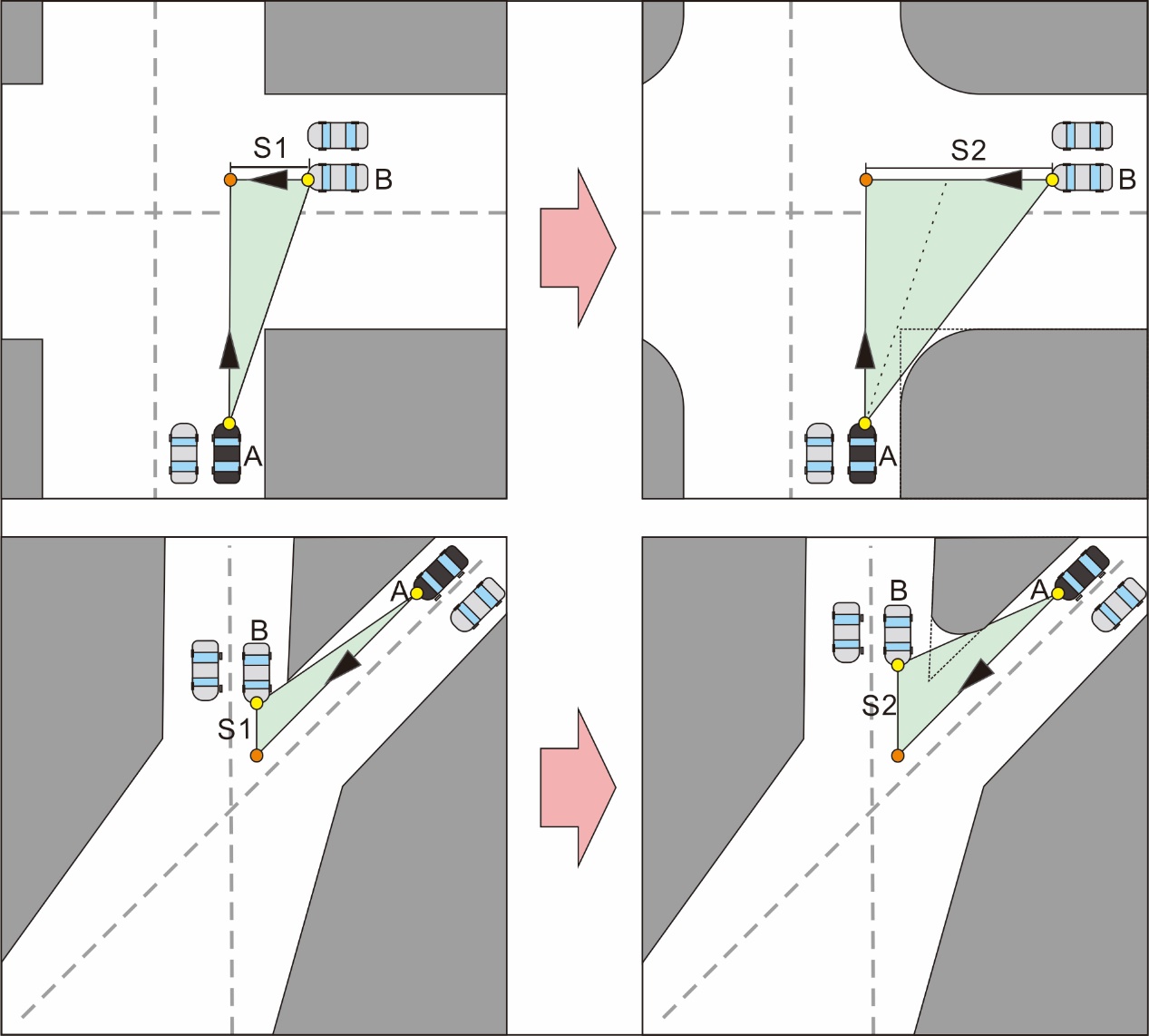


Figure S2. Curved corners improve driver confidence when cornering or going straight (cf., Easa, 2000).


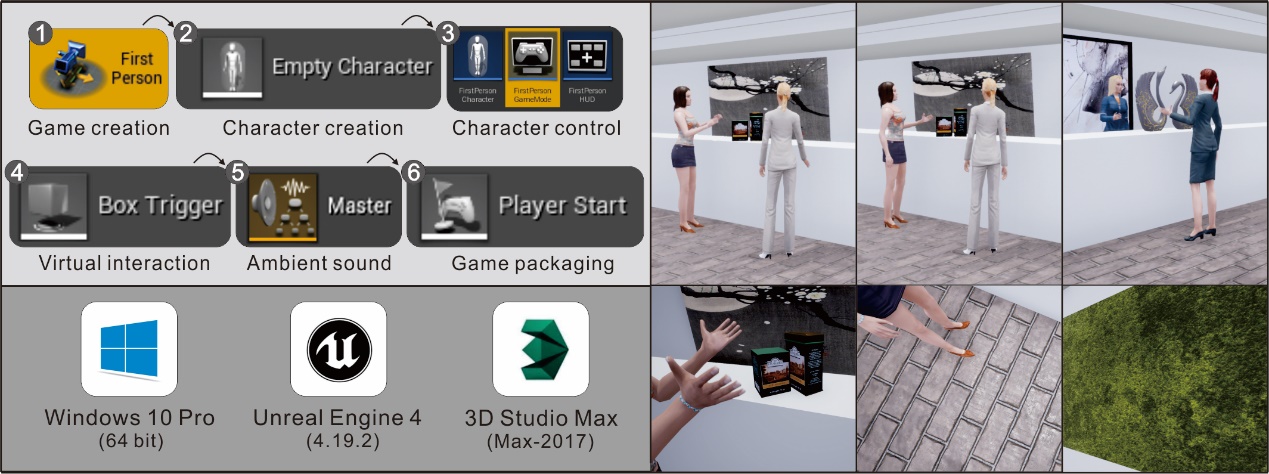
Figure S3. Details of the operational procedures.


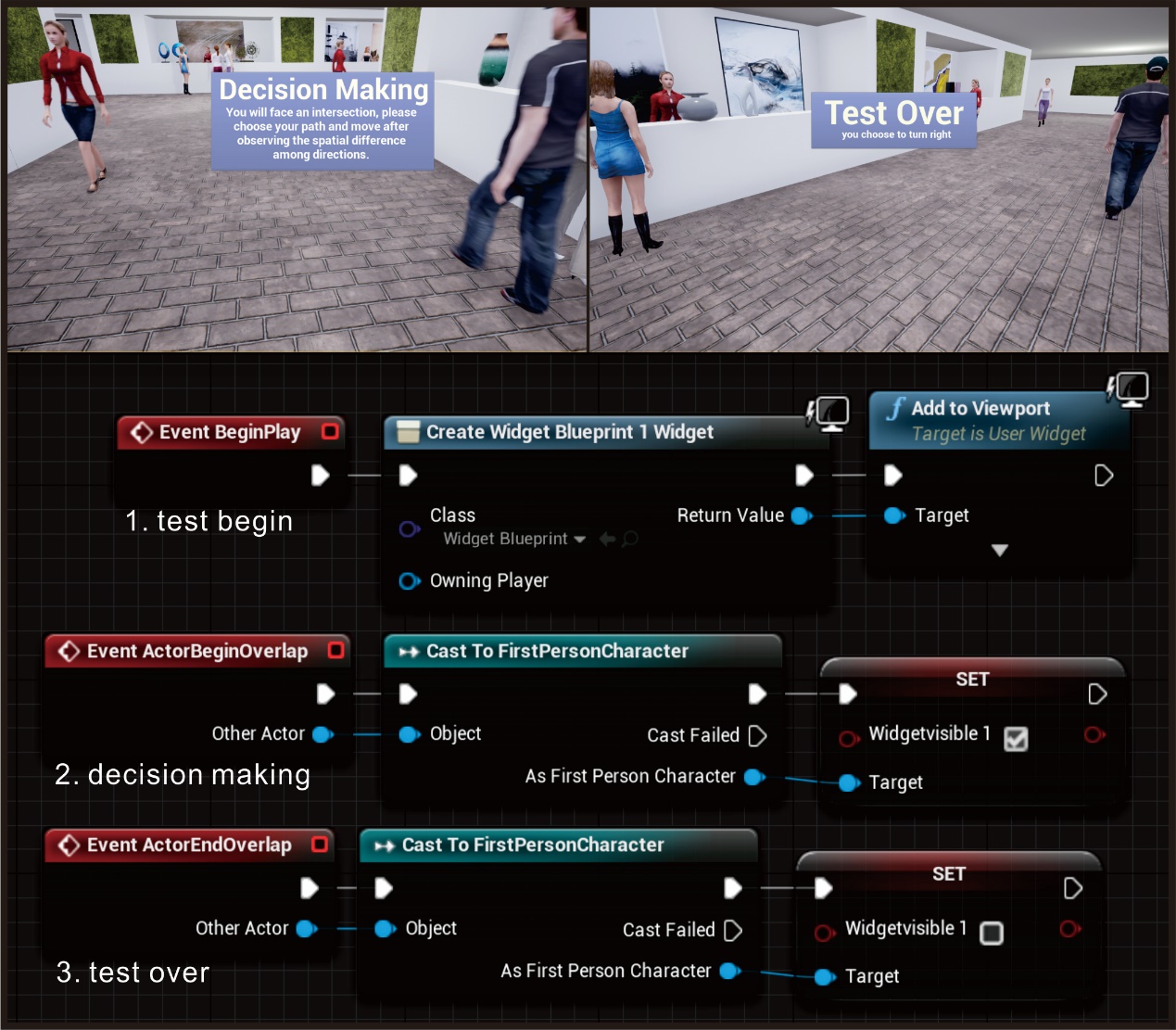
 Figure S4. Blueprint code used to guide the wayfinding simulation.


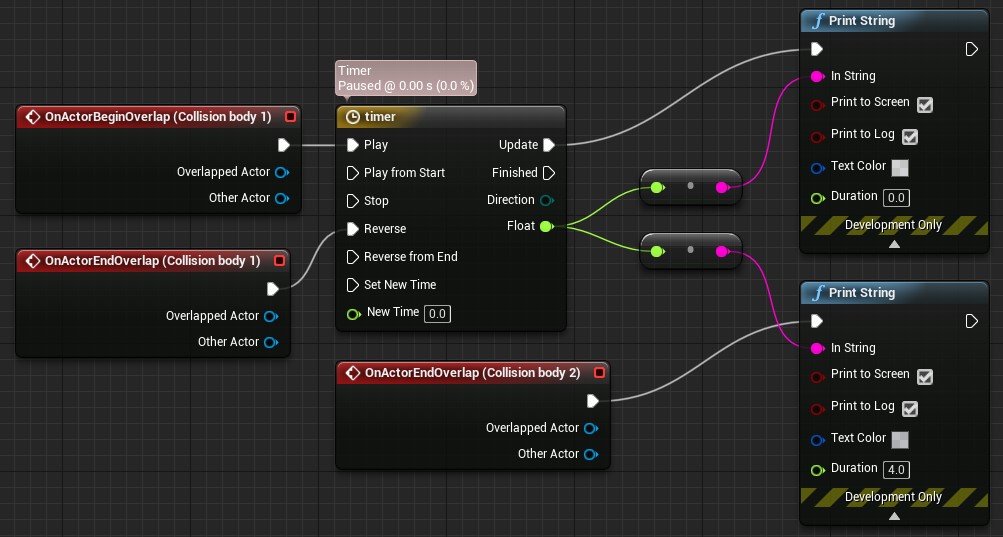


Figure S5. Blueprint timing function code of UE4.


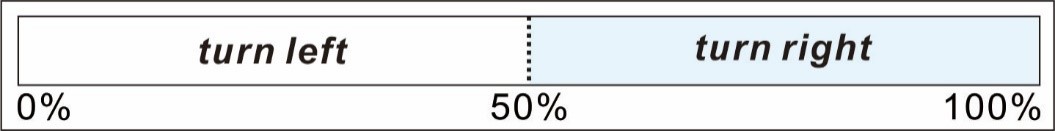


Figure S6. 50% is a theoretical threshold between left- and right-turn performance (Raubal and Egenhofer, 1998).


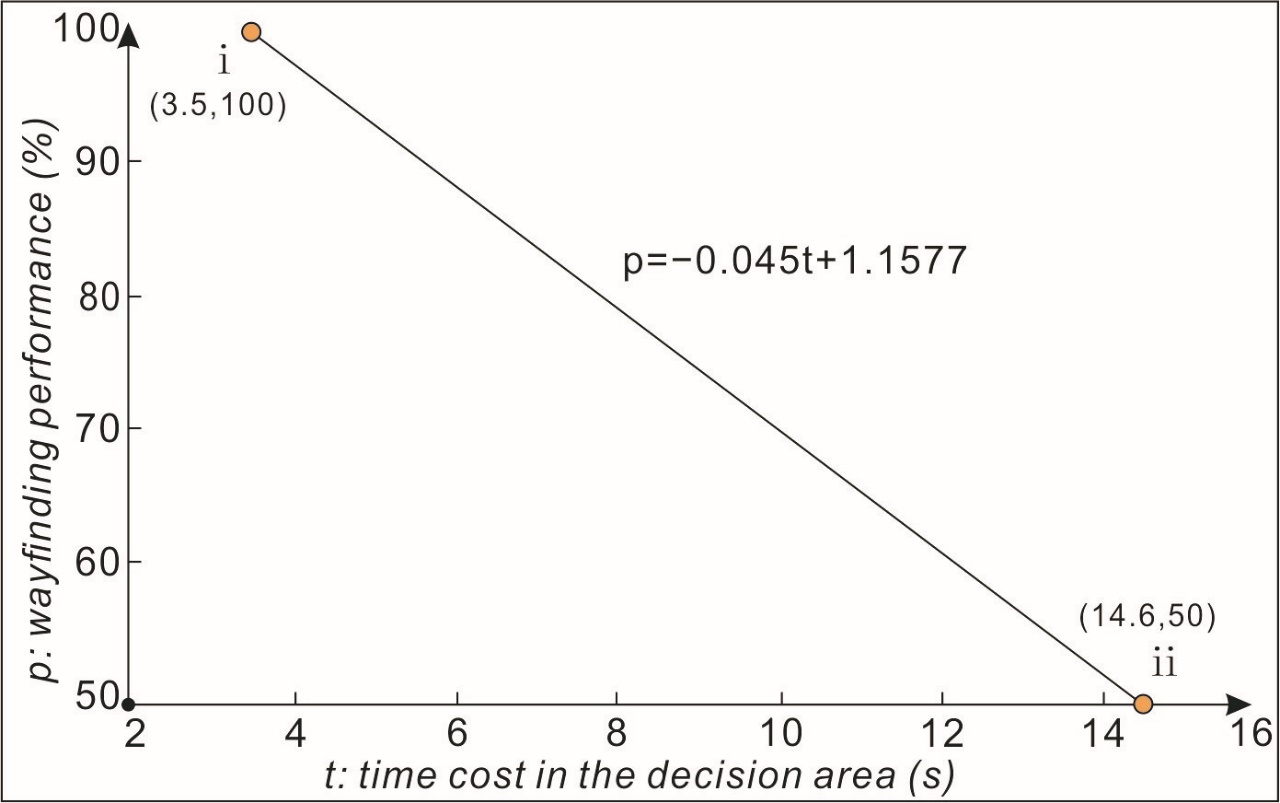


Figure S7. Time cost versus right-turn performance.
